# Erk1/2 is not required for endothelial barrier establishment despite its requirement for cAMP-dependent Rac1 activation in heart endothelium

**DOI:** 10.1080/21688370.2024.2398875

**Published:** 2024-09-04

**Authors:** Sina Moztarzadeh, Hilda Vargas-Robles, Michael Schnoor, Mariya Y. Radeva, Jens Waschke, Alexander Garcia-Ponce

**Affiliations:** a Chair of Vegetative Anatomy, Faculty of Medicine, Ludwig-Maximilians-University (LMU) Munich, Munich, Germany; b Department of Molecular Biomedicine, Center for Research and Advanced Studies of the National Polytechnic Institute (CINVESTAV-IPN), Mexico City, México

**Keywords:** Adherens junctions, cAMP, Erk1/2, MAPK, Rac1, Endothelial cells, GTPases, Tight junctions, Transendothelial electrical resistance, Vascular permeability

## Abstract

The contribution of Erk1/2 to endothelial barrier regulation is convoluted and differs depending on the vascular bed. We explored the effects of Erk1/2 inhibition on endothelial barrier maintenance and its relationship with cAMP-dependent barrier strengthening. Thus, myocardial endothelial cells (MyEnd) were isolated and protein expression, localization and activity of structural and signaling molecules involved in maintenance of endothelial function were investigated by Western blot, immunostainings and G-LISA, respectively. The transendothelial electrical resistance (TEER) from confluent MyEnd monolayers was measured and used as a direct indicator of barrier integrity in vitro. Miles assay was performed to evaluate vascular permeability in vivo. Erk1/2 inhibition with U0126 affected neither the structural organization of adherens or tight junctions nor the protein level of their components, However, TEER drop significantly upon U0126 application, but the effect was transitory as the barrier function recovered 30 min after treatment. Erk1/2 inhibition delayed cAMP-mediated barrier strengthening but did not prevent barrier fortification despite diminishing Rac1 activation. Moreover, Erk1/2 inhibition, induced vascular leakage that could be prevented by local cAMP elevation in vivo. Our data demonstrate that Erk1/2 is required to prevent vascular permeability but is not critical for cAMP-mediated barrier enhancement.

## Introduction

The endothelium is the cellular layer lining of the inner surface of blood vessels and thereby acts as a key player in many biological processes such as the formation of new blood vessels, blood flow, vascular permeability, and the immune response.^1^ Many of these processes involve the activation of the mitogen-activated protein kinases (MAPK)/extracellular signal-regulated kinase 1/2 (Erk1/2) pathway.^2^ However, very little is known about the involvement of Erk1/2 in endothelial junction and barrier regulation. Published studies show controversial data: while some indicate that Erk1/2 is beneficial for endothelial barrier function; others describe detrimental properties. For instance, endothelial-specific induced deletion of Erk2 in 12–16-week-old Erk1-KO mice was fatal, and these animals exhibited important heart pathophysiological features, including intraventricular conduction delays to atrial flutter, complete heart block, and myocardial fibrosis,^3^ indicating the requirement of Erk1/2 for proper endothelial functions in heart. Conversely, SCH772984-mediated inhibition of Erk1/2 in primary human lung microvascular endothelial cells (HMVEC) enhanced lipopolysaccharide (LPS)-induced interleukin-6 production and prevented endothelial hyperpermeability, suggesting that Erk1/2 contributes to toll-like receptor 4 (TLR4)-dependent endothelial vascular leakage.^4^ The controversial outcomes in those studies can be partly explained by the heterogeneity of endothelial cells studied which were derived from different vascular beds.^5^ Therefore, further investigations are required to shed more light on endothelial Erk1/2 functions.

Besides Erk1/2, other signaling cues govern endothelial barrier stability. Particularly, the second messenger cyclic adenosine monophosphate (cAMP), known to exert barrier enhancing effects on endothelial cells,^6^ was linked to the regulation of Erk1/2 via protein kinase A (PKA). The pharmacological inhibition of cAMP-mediated activation of PKA triggered Erk1/2 activity via its upstream activator Raf-1, increasing paracellular gap formation and actin stress fiber formation in bovine pulmonary artery endothelial cells.^7^ This finding highlights a connection between the cAMP pathway, Erk1/2, and the actin cytoskeleton that contributes to endothelial barrier regulation. Nevertheless, it is still unclear how the structure of cell junctions is affected by Erk1/2 inhibition in combination with cAMP elevation. On the other hand, it is well known that cAMP promotes both Rac1 activation and RhoA inhibition.^6,8^ A cross-talk between Erk1/2 and these GTPases has also been suggested. For instance, Erk1/2 signaling induces endothelial cell angiogenesis and antagonizes activation of Rho-associated coiled-coil containing protein kinase (ROCK) and myosin light chain (MLC) phosphorylation.^9^ Moreover, inhibition of ROCK with H-1152P leads to increased vascular endothelial growth factor (VEGF)-mediated Erk1/2 activation,^10^ suggesting that these pathways are mutually exclusive in endothelial cells. Thus, neither the function of Erk1/2 or its modulation are quite the same in all cell types and tissues, limiting the opportunities for the use of systemic therapeutic treatments. Therefore, it is of utmost importance to better understand selective Erk1/2 signaling in each particular tissue or vascular bed.

Here, we found that in MyEnd cells, U0126-mediated Erk1/2 inhibition affects endothelial barrier function without inducing changes in the composition or distribution of adherens junctions (AJ) or tight junctions (TJ). Inhibition of Erk1/2 with U0126 did not prevent cAMP-mediated barrier fortification mediated by F/R treatment. The data show that Erk1/2 is indeed relevant to preserve the vascular barrier but also reveal that Erk1/2 functions may also be divergent in endothelial cells from different origins.

## Materials and methods

### Animals, endothelial cells isolation animal ethical approval

For *in vivo* vascular permeability assessment, 8–12 weeks old C57BL/6 male mice were obtained from the animal facility at CINVESTAV, Mexico City, Mexico. All protocols have been approved by the institutional animal care and use committee of CINVESTAV (Protocol number: 0263–18). Animals were transported to the laboratory inside Super Mouse 750™ ventilated cages and were kept inside until needed. Food and water were provided ad libitum. A maximum of 5 animals per cage were housed under normal atmospheric conditions.

For *in vitro* experiments, MyEnd were isolated from heart tissues obtained from C57BL/6J mice hosted in our local animal facility (LMU-Munich, Germany). The handling of the mice and the relevant protocols were approved by the Ethics committee of the Regierung von Oberbayern, Germany (Gz. 55.2-1-54-2532-139-2014). All methods were performed in accordance with the relevant guidelines and regulations. Dr Mariya Y. Radeva and Dr Alexander Garcia-Ponce performed the isolation of the cells. 2–4 days old newborn mice were used. The pups were sacrificed by decapitation without anesthesia. A small piece of the tail was collected and used for genomic DNA extraction and genotyping. Subsequently, the hearts were extracted, chopped into small fragments and digested with trypsin (0.05%)-collagenase A (0.02%) solution for at least 2 h at 37°C, vigorous shaking was done every 15 min. The digestion was terminated by adding an equal volume of ice-cold buffer (153 mM NaCl, 5.6 mM KCl, 2.3 mM CaCl_2_ × 2 H_2_O, 2.6 mM MgCl_2_ × 6 H_2_O, 15 mM HEPES, 1% BSA). After centrifugation, the supernatants were gently removed and the pellets resuspended in Dulbecco’s Modified Eagle’s medium (DMEM, Gibco-Thermo Fisher, #41966-029), supplemented with 50 U/ml Penicillin G/Streptomycin (Sigma Aldrich Chemie GmbH, Taufkirchen, Germany) and 10% Fetal calf serum (FCS, Biochrom, #S0115/0247X). Isolated cells were cultivated on gelatine-coated dishes and grown under standard cell culture conditions (37°C, 5% CO2 with saturating humidity). A day after, adherent cells were incubated with Polyoma virus middle T antigen secreted by GP+E-86 Neo (GPENeo) fibroblast and thereby, the growth of endothelial cells over nonendothelial cells was promoted. After 4–6 weeks of culturing, homogeneous endothelial cell monolayer was obtained. The endothelial markers von Wilebrand Factor (vWF), PECAM-1 and VE-cadherin were used to confirm the cells’ phenotype as published previously.^11–13^

### Mediators and antibodies

Induction of intracellular cAMP was achieved by application of 5 μM Forskolin (Sigma Aldrich Chemie, #F6886) and 10 μM Rolipram (Sigma Aldrich Chemie, #R6520). Blocking MEK1/2-Erk1/2 activity was done with the specific inhibitor U0126 at a final concentration of 10 μM (Cell Signaling, 9903S). Vascular permeability *in vivo* was induced by treatment with 450 ng of histamine (Sigma Aldrich Chemie, #53300). The following antibodies were used: VE-cadherin (Abcam, ab33168), β-catenin (BD Transduction Laboratories 61,054), claudin-5 (Abcam, ab15106), ZO-1 (Termo Fisher Scientific 617,300), Erk1/2 (Cell Signaling, 9102), pErk1/2 (Cell Signaling, 4370S), all used at 1:1000 dilution; α–tubulin (Abcam, ab7291), used at 1:2000 for Western blot.

### Western blot

After splitting, 2 × 10^5^ cells were seeded on gelatine-coated 6-well plates and grown until confluency. Plates were placed on ice and washed with ice-cold PBS. Whole-cell lysates were obtained using SDS-lysis buffer (25 mM HEPES, 2 mM EDTA, 25 mM NaF and 1% SDS, pH 7.6) added with cOmplete™ protease inhibitor cocktail (Roche Diagnostics, #11697498001) and PhosStop EASYpack (Roche Diagnostics, #4906845001). Cell lysates were sonicated and centrifuged at 14,000 rpm at 4°C for 1 min, right after, the supernatants were collected and transferred to fresh pre-cooled tubes. Protein concentration was estimated using the BCA standard colorimetric assay (Thermo Fischer Scientific, #23225). Subsequently, samples were mixed with 3 × Laemmli buffer (1:1) and boiled for 5 min at 95°C to denatured the proteins, which subsequently were separated by SDS-PAGE and transferred onto nitrocellulose membranes (Thermo Fischer Scientific, #LC2006), with a pore size of 0.45 µm. Membranes were blocked with tris-buffered saline containing 5% bovine serum albumin (BSA) and 0,1% Tween (TBS-T/BSA) for 1 h at room temperature. Following this step, the membranes were incubated with the primary antibodies of interest overnight at 4°C on a rocker. After several washing steps with TBS-T, the membranes were incubated with horse-radish-peroxidase-(HRP)-species-specific secondary antibodies at room temperature for 1 h. Unbound secondary antibodies were washed out with TBS-T and the proteins of interest were visualized using the Amersham Imager 600 (GE Healthcare, AI600). Pixel intensity quantification from SDS-PAGEs was performed using ImageJ (NIH, Windows version 64-bit).

### Immunofluorescent staining

For this experiment, 1.2 × 10^5^ MyEnd were grown on 12 mm glass coverslip coated with 5% gelatine. Confluent monolayers were fixed with 4% paraformaldehyde for 10 min at room temperature and permeabilized with 0.1% Triton-X-100 diluted in PBS for 5 min. Unspecific antibody binding was blocked by incubation with a mixture of 1% BSA (VWR, #422351S) and 10% normal goat serum (Thermo Fisher Scientific, # 31872) for 20 min at room temperature. The proteins of interest were immunolabelled with primary antibodies for 1 h at room temperature. After several washes with PBS, cells were incubated with species-specific Cy3-labeled secondary antibodies (1:200). After several washes with PBS, the monolayers were rinsed with H20 as a final step. The coverslips were mounted on microscope glass slides with self-made antifade medium. The images were captured with a laser scanning confocal microscope (Leica SP5) equipped with a HCX PL APO Lambda blue 63 × 1.4 oil immersion objective (Leica).

### Transendothelial electrical resistance (TEER)

Endothelial barrier function *in vitro* was measured with the ECIS Z Theta system (Applied Biophysics). Briefly, 6.0 × 10^4^ cells were seeded on gelatine-coated 8W10E gold arrays (Ibidi, #72010). TEER was measured every 5 min, using the multi-frequency (MTF), the frequency of 4000 Hz was determined as the best to evaluate MyEnd’s barrier dynamics. All TEER data are presented as fold-change, normalized to the initial point of measurement.

### G-LISA

The activation state of small GTPases was assessed using the Rac1 and RhoA G-LISA kits (Cytoskeleton, #BK128 and #BK124, respectively). The experiments were conducted according to the manufacturer’s instructions. The absorbance was measured at 490 nm using the TECAN Infinite 200 PRO device.

### Miles Assay

To measure vascular permeability *in vivo*, Evans Blue dye was used as previously described.^14^ Briefly, vascular permeability in the skin was evaluated by injecting 8–12 weeks old C57BL/6J male mice intraperitoneally with 2% Evans Blue dye in saline solution (4 ml/kg of body weight). After 2 h, mice were anesthetized intraperitoneally by applying a mixture of ketamine/xylazine (100 mg/kg and 13 mg/kg of body weight, respectively) in 0.9% saline solution. The back skin fur was carefully shaved with a veterinary clipper shaver. A drop of 0.9% saline solution is administered on each eye to avoid them from drying. Consequently, 50 µl from each of the analyzed substances were intradermally injected into the back skin. Afterwards, all animals were transferred to their cages. After 30 min, mice were sacrificed by cervical dislocation, and the skin around the injection site was excised (circles of 1 cm in diameter) and weighted. The tissue circles were immersed separately in formamide for 24 h to extract the blue dye and then read spectrophotometrically at 620 nm. Values were expressed as the ratio between the optical density (O.D.) and tissue weight (mg).

### Pixel intensity quantification

Confocal Z-stacks images were analyzed with the FIJI (enhanced ImageJ) software. A small area surrounding the junctions was drawn using the “rectangle” tool. The integrated density was recorded from at least 120 randomly selected junctions, coming from four independent experiments. The values were averaged and expressed as arbitrary units (A.U.).

### Statistical analyses

The graphs supporting the current study were generated with the Prism Software version 8 (Graph pad). The data are presented as mean ± SD. To compare the effect between only two groups, unpaired two-tailed Student T-test was employed. To test the significance among various treatments at a certain time, one-way analysis of variance (ANOVA) was applied. To statistically compare the different treatments at various time-points, Two-way ANOVA followed by Turkey multiple comparison test was used. Values equal or below 0.05 (*); 0.01 (**); 0.001 (***) and 0.0001 (****) were considered statistically significant.

## Results

### Inhibition of Erk1/2 induces a transitory drop of barrier resistance but does not prevent cAMP-mediated barrier strengthening

**Figure 1.**
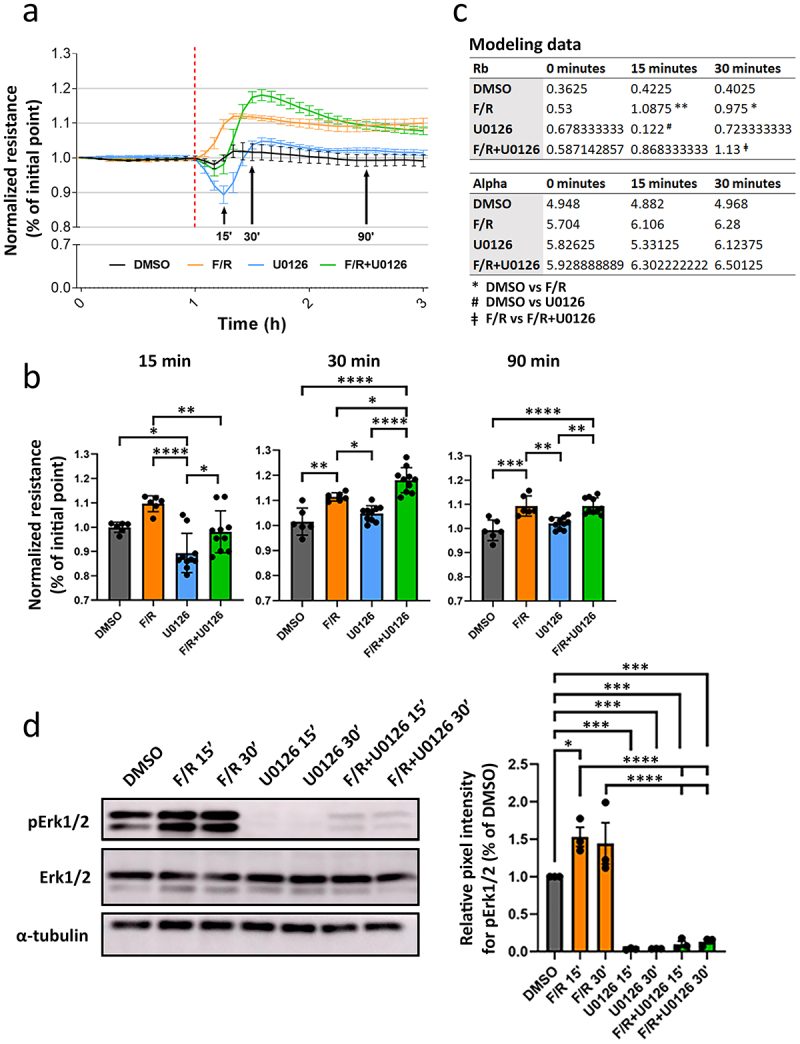
Effect of Erk1/2 inhibition on endothelial barrier and cAMP-mediated barrier enhancement. (a) barrier function measurements (TEER). Data are normalized to the point of start for each respective condition. The red-dotted line indicates the time where the treatments were applied. The arrows indicate time-points of interested where the differences between the conditions were statistically analysed, *N* = 6. (b) bar graphs showing the significant differences between the treatments 15, 30 and 90 minutes after application, *N* = 6. (c) modelling data calculated using the built-in ECIS software “Model” tool using the TEER values recorded in MTF mode. Absolute values ± S.D. are shown. (d) Representative Western blots demonstrating the phosphorylation of Erk1/2 after treatment. α-tubulin is utilized as a loading control. The bar graph depicts the pixel intensity from the bands of interest normalized to the DMSO control *N* = 3. Data are presented as mean ± S.D.*p < 0.05; **p < 0.01; ***p < 0.001; ****p < 0.0001.

We examined the effects of Erk1/2 inhibition on barrier function of confluent MyEnd monolayers by measuring TEER. While DMSO-treated cells showed a stable resistance for the duration of the experiment (Figure 1(a,b), black line-bar), cells exposed to U0126, an Erk1/2 inhibitor, exhibited a fast and significant drop of barrier resistance, 15 min after application. The decrease in TEER was recovered 30 min post-treatment (Figure 1(a,b), blue line-bar). As expected, induction of intracellular cAMP by F/R led to a rapid and sustained increase of TEER (Figure 1(a,b), orange line-bar). Simultaneous application of U0126 and F/R led to a delay in barrier strengthening compared to F/R treatment alone (Figure 1(a,b), green line-bar). Surprisingly, 30 min after application, the resistance of cell monolayers treated with F/R+U0126 was significantly higher than that observed for the treatment with F/R only. This effect was lost before the 90-min mark, at which point, the TEER values from F/R and F/R+U0126 were comparable. Moreover, the aforementioned effects were specific to the paracellular current flux and not a side-effect caused by changes in cell-to-surface adhesion by modeling the parameters “Rb” and “alpha”, referring to the paracellular space and the basal adhesion of endothelial cells, respectively (Figure 1(c)). As next, we evaluated the phosphorylation of Erk1/2 (pERK1/2), used as a readout for its activation. Western blot analyses revealed that F/R-mediated elevation of intracellular cAMP concentration increased Erk1/2 phosphorylation MyEnd. Conversely, treatment with U0126 almost completely abolished this effect and significantly impaired Erk1/2 activation via F/R application and thus confirmed that U0126 efficiently blocks Erk1/2 activity in all experiments performed (Figure 1(d)).

### Cell contacts integrity and the expression of junctional proteins are not affected by Erk1/2 inhibition

**Figure 2.**
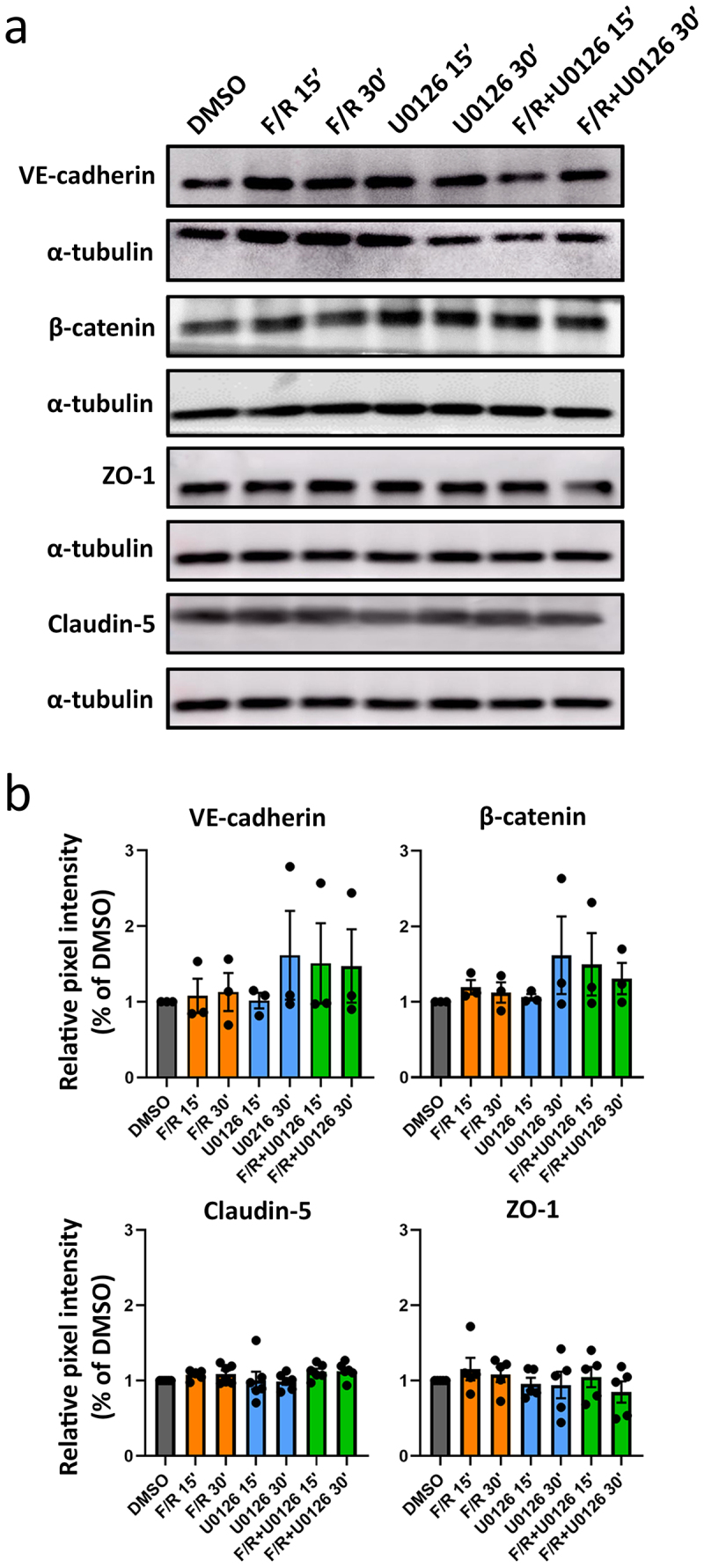
Protein pattern of the main endothelial AJ and TJ components following Erk1/2 inhibition and/or cAMP stimulation. (a) Representative Western blots for critical junctional proteins from at least three independent experiments. α-tubulin is used as loading control. (b) bar diagram representing the quantified pixel intensity from the blots presented in (a) normalized to DMSO, *N* = 3-5. Data are presented as mean ± S.D.

**Figure 3.**
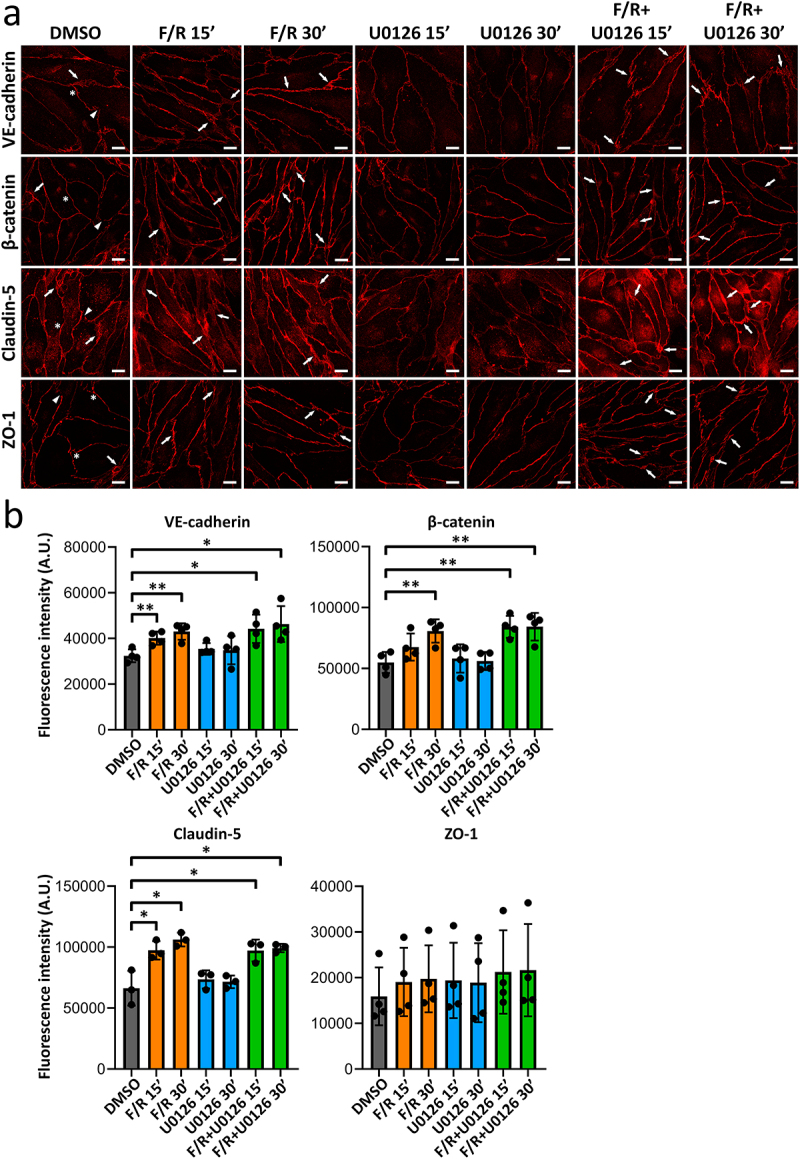
Endothelial junctional organization after blocking Erk1/2 function with or without F/R-mediated cAMP increase. (a) Representative Z-stack confocal images showing the membrane distribution of ve-cadherin, β-catenin, claudin-5 and ZO-1 after treatments. The white scale bar equals 10 µm. For DMSO, stars display continuous junctions, arrowheads point to areas where no membrane signal is visible and arrows indicate reticular/thick contacts. For all other conditions, arrows show reticular/thick cellular unions *N* = 3-4. (b) bar diagram, representing the pixel intensity quantification from at least three independent experiments. The values are presented as mean ± S.D. with arbitrary units (A.U.). Scale bar equals 10µm. *p < 0.05; **p < 0.01.

We investigated whether the observed changes in TEER were due to alterations in the abundance of AJ and TJ composition. Western blots and pixel intensity quantifications revealed that treatment with U0126 or F/R had no significant effect on the protein levels of VE-cadherin and β-catenin or claudin-5 and ZO-1, classical components of adherens and tight junctions, respectively (Figure 2(a,b)). Using laser scanning confocal microscopy, we also explored the localization patterns of those junctional molecules. Z-stack images showed that the distribution of the proteins in control monolayers was mostly continuous, with occasional segmented junctions and reticular regions, defined as areas where the cells overlap^15^ (Figure 3(a), stars, arrowheads and arrowheads, respectively). Treatment with F/R increased the appearance of reticular zones (Figure 3(a), stars) and membrane signal intensity of VE-cadherin and claudin-5 after 15 min as revealed by pixel intensity quantification (Figure 3(b), bar graph). The effects remained 30 min after treatment, where a significant increase in signal intensity for β-catenin was also denoted (Figure 3(b), bar graph). cAMP elevation did not affect the signal intensity of ZO-1. To our surprise, despite reducing TEER (Figure 1), inhibition of Erk1/2 had no major influence on the distribution or signal intensity of any of the proteins investigated (Figure 3(a,b)). In addition, cells simultaneously treated with F/R and U0126 displayed junctional patterns similar to those observed with F/R treatment alone, where more often reticular regions were spotted (Figure 3(a), arrows), an effect paralleled with increased signal intensity for VE-cadherin, β-catenin and claudin-5 at the plasma membrane (Figure 3(a), bar graph). Collectively, the data demonstrate that Erk1/2 does not regulate barrier strengthening via cell–cell contact reorganization (Figure 3(a,b)).

### The establishment of heart endothelial barrier is independent of Erk1/2

**Figure 4.**
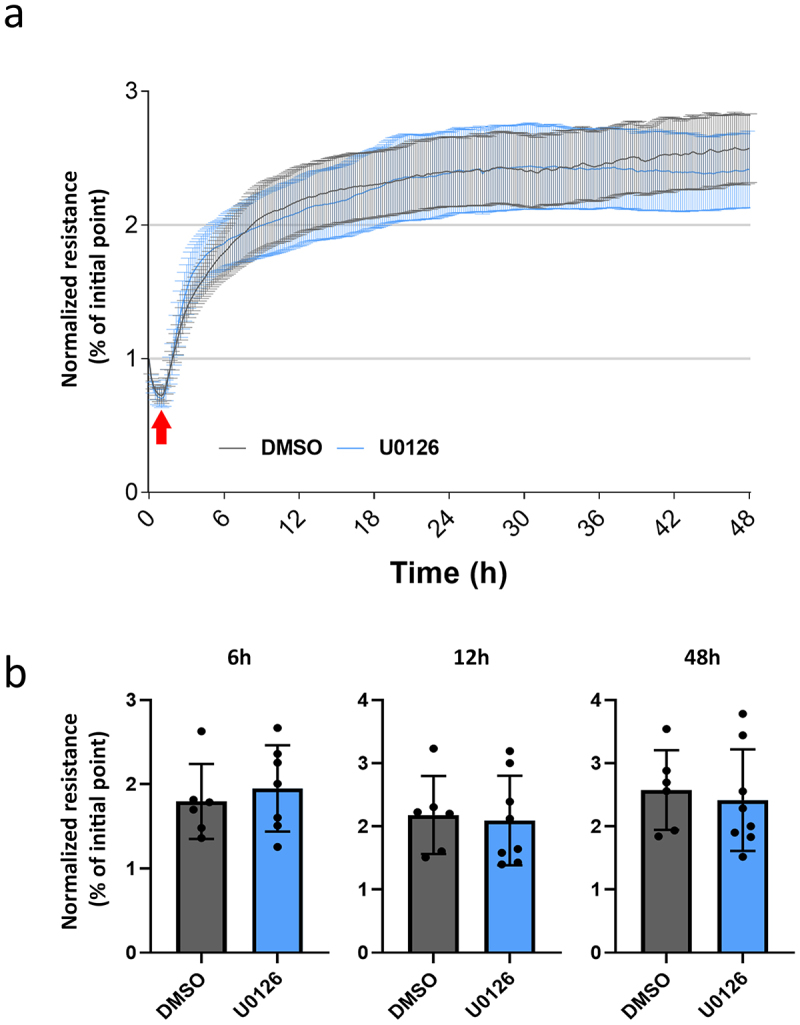
Effect of Erk1/2 inhibition on endothelial barrier establishment. (a) MyEnd were seeded on ECIS arrays and grown from the start with medium containing either DMSO or U0126 (indicated by the red arrow). TEER was recorded for 48 h. Data are normalized to the initial point of measurement. *N* = 4. (b) bar graphs showing the TEER values in U0126 treated monolayers, relative to DMSO, at the indicated time points.

Given the previous results, we hypothesized that Erk1/2 is more relevant for the formation of the endothelial barrier, rather than its maintenance. To explore this hypothesis, MyEnd seeded on gelatine-coated ECIS arrays, were cultivated in medium containing either U0126 or DMSO as a control, and TEER was measured. Unexpectedly, pharmacological inhibition of Erk1/2 did not hinder barrier establishment over time (Figure 4a,b), blue line and bar, respectively). After reaching stable TEER (around 18 h post-seeding), MyEnd treated with U0126 maintained a normal functional barrier, similarly to the control cells (Figure 4(a,b), blue and black line, respectively). No major differences in TEER were observed during the 48-h duration of the experiment.

### cAMP-induced activation of Rac1 is abolished by Erk1/2 inhibition

**Figure 5.**
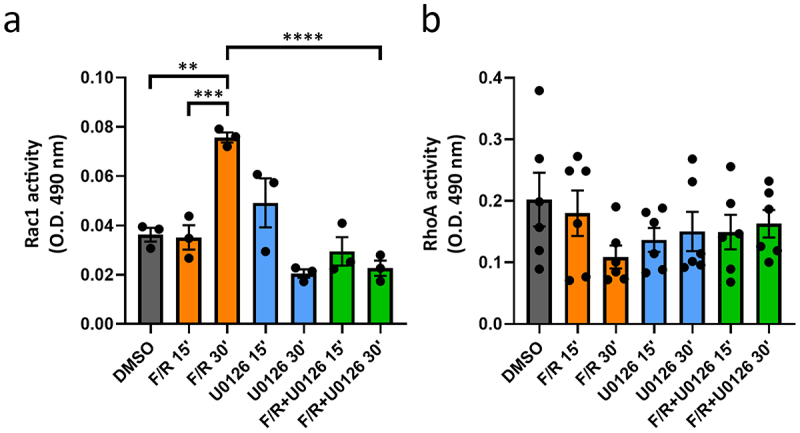
Modulation of small GTPases activity by U0126 and F/R. (a) activation level of Rac1 determined by G-LISA, *N* = 3. (b) activation of RhoA, *N*=6. Raw data are presented as the mean ± S.D. of O.D. **p < 0.01; ***p < 0.001; ****p < 0.0001.

Among the signaling cascades triggered by cAMP, small GTPases such as Rac1, and RhoA, are important downstream targets relevant for endothelial barrier regulation.^6^ Therefore, we investigated the effect of Erk1/2 inhibition alone or in combination with F/R-mediated cAMP elevation on Rac1 and RhoA activity. Using G-LISA assays, we found that treatment with F/R induced a significant activation of Rac1 only after 30 min (Figure 5(a)). In contrast, inhibition of Erk1/2 had no major impact on the activation of Rac1 independently of the time of treatment. Surprisingly, U0126 application alone significantly impaired F/R-induced Rac1 activation after 30 min, indicating that Erk1/2 is required for cAMP-mediated activation of Rac1. On the other hand, we did not observe any alterations in the activity of RhoA in any of the conditions tested (Figure 5(b)), suggesting that RhoA activation is not related to the TEER drop caused by Erk1/2 inhibition.

### Erk1/2 inhibition induces vascular hyperpermeability in vivo, an effect prevented by cAMP elevation

**Figure 6.**
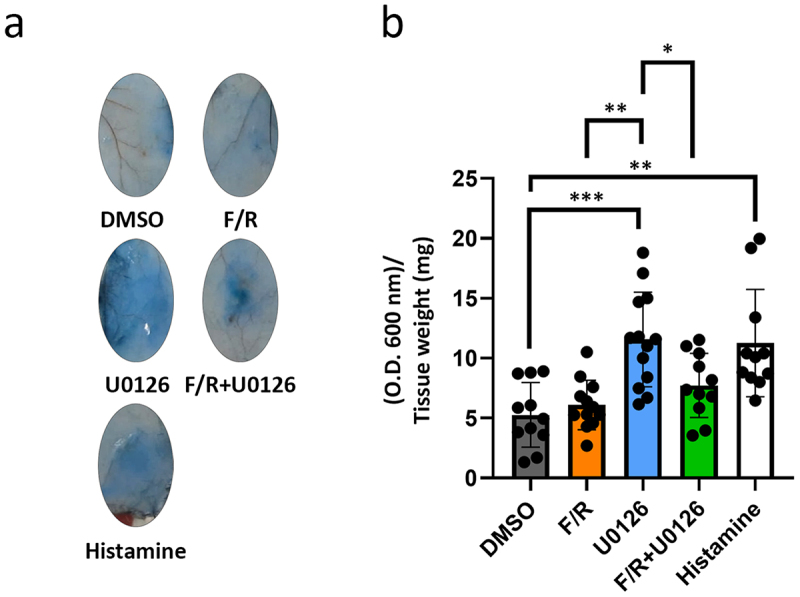
Analysis of perivascular permeability *in vivo*. (a) Representative images from freshly isolated skin sections obtained shortly after Miles assay. *p < 0.05; **p < 0.01; ***p < 0.001. b) bar diagram depicting the skin vascular leakage of Evans blue due to different treatments. The values are expressed as the ratio between absorbance and tissue weight ± S.D. of O.D., *N* = 11-13.

Our *in vitro* data using MyEnd showed that Erk1/2 is partially involved in the regulation of endothelial barrier functions. Thus, by using Miles assay to measure vascular permeability in the skin, we investigated whether this is also true *in vivo*. These experiments revealed a similar Evan’s blue leakage ratio (O.D. 620 nm/tissue weight) between DMSO and F/R (Figure 6(b), gray and orange bars, respectively). Treatment with U0126 alone led to significantly higher vascular permeability (Figure 6(b), blue bar) similar to what we observed *in vitro* (Figure 1). Interestingly, the increased leakage ratio induced by U0126 was comparable to that obtained with histamine, a permeability-inducing substance (Figure 6(b), white bar). Importantly, the administration of F/R together with U0126 prevented the permeability-inducing effect of Erk1/2 inhibition (Figure 6(b), green bar). Together, our data indicate that Erk1/2 plays a critical role in endothelial barrier function preservation *in vivo*.

## Discussion

Hitherto, the role of Erk1/2 in controlling endothelial barrier function is controversial and poorly understood. In this study, we aimed to better understand the function of Erk1/2 in endothelial junction regulation and cAMP-mediated barrier enhancement. To this end, we explored the effects of Erk1/2 inhibition and found that inhibiting Erk1/2 decreased barrier function *in vitro* and *in vivo*, indicating that Erk1/2 is necessary to maintain barrier integrity in mature endothelial cells. This finding is in line with previous studies showing that Erk1/2 is required for embryonic development, endothelial barrier functions, and organ homeostasis. For instance, endothelial-specific deletion of Erk1 and Erk2 in mice led to embryonic lethality,^16^ and adult Erk1-KO mice with a tamoxifen-induced endothelial-specific Erk2 depletion (Erk1/EC-Erk2-KO) developed vascular anomalies in different organs, ranging from organ hemorrhage to capillary cell membrane loss and delamination. Importantly, all Erk1/EC-Erk2-KO mice died within 5 weeks after tamoxifen injection.^3^ Here, we also observed that despite Erk1/2 inhibition, MyEnd barrier function quickly reverted back to control levels showing that although Erk1/2 is important for barrier homeostasis, the effects are transient and endothelial cells regain barrier function independently of Erk1/2. In our model, F/R application induces cAMP-mediated barrier strengthening and Erk1/2 activation (Figure 1), falling in line with a previous study.^17^ Therefore, the data hint for an interplay between Erk1/2 and cAMP in respect of barrier strengthening. When confluent MyEnd were cotreated with F/R and U0126, barrier strengthening was delayed by approximately 15 minutes, thus matching the resistance drop caused by U0126 alone. Despite this short delay, resistance enhancement still occurred and was, for a short period, higher than the one observed in cells treated with F/R alone. However, around 90 minutes after treatment, both conditions showed similar TEER fold-changes. In accordance with this finding, HUVECs treated with the Epac agonist 8-pCPT-2′-O-Me-cAMP in combination with U0126 and thrombin had reduced endothelial permeability.^17^ Moreover, blocking Erk1/2 protected against lipopolysaccharide-induced lung injury.^18^ Together, our data and these studies indicate that Erk1/2 does not play a critical role in cAMP-mediated endothelial barrier stabilization. This idea is further supported by Western blot and immunostainings of endothelial junction proteins. Treatment with F/R, U0126 or the combination of both had no significant impact on the protein levels of VE-cadherin, β-catenin, claudin-5 and ZO-1 (Figure 2). In addition, the junctional localization and cAMP-mediated junctional accumulation of VE-cadherin, β-catenin and claudin-5 were unaffected by Erk1/2 inhibition (Figure 3). It is well-accepted that activation of Erk1/2 is associated with angiogenesis, improved endothelial survival, migration, and proliferation.^16,17^ Taken this into account, we speculated that interfering with Erk1/2 activity in growing endothelial cells would have an impact on barrier establishment. Surprisingly, freshly seeded MyEnd treated with U0126 were able to form a functional endothelial barrier similar to control cells (Figure 4). This finding indicates that MyEnd do not require Erk1/2 to grow efficiently and can develop a functional barrier. The results are, however, not surprising as many different mechanisms are engaged to facilitate barrier formation, including the activation of small GTPases and reorganization of the actin cytoskeleton.^2^ In this regard, we also investigated the contribution of two GTPases known to modulate endothelial barrier function, Rac1 and RhoA.^19^ Our data showed that RhoA activation was not affected by any of the treatments. By contrast, F/R treatment alone induced a significant increase of Rac1 activation that was abolished by cotreatment with U0126. In line with these findings, HUVECs pre-treated with U0126 and subjected to hypoxia also exhibited reduced Rac1 activation.^20^ In pulmonary microvascular endothelial cells (PMVECs) high mobility group box 1 (HMGB1) led to a dose and time-dependent increase of Rac1 expression and activation, concomitant with increased Erk1/2 activity. This effect was partially blocked by a Rac1-specific siRNA.^21^ Collectively, these reports and our data suggest that a link between Rac1 and Erk1/2 exists. The complex nature of Erk1/2-Rac1 regulation reported here and its effects on endothelial barrier function could be a direct consequence of the fine-tuned spatial and temporal modulation of small GTPases, implicating guanine nucleotide exchange factors (GEFs), GTPase-activating proteins (GAPs) and guanosine dissociation inhibitors (GDIs) (recently reviewed in^22^). For example, U0126 could restrict the cytoplasmic activation of Rac1 without having a direct impact on Rac1 pools localized at cellular contacts. This could explain in part, why barrier enhancement was observed despite the lack of significant total Rac1 activation in cells treated with both F/R and U0126 simultaneously. In this context, the Rac1 GEF Tiam1 is an attractive subject of investigation, known to reinforce endothelial barrier locally at the membrane.^23^ In addition, inhibition of Erk1/2 may trigger a compensatory reaction to preserve endothelial barrier that coexists with barrier destabilization. For example, it could involve the small GTPase Cdc42, reported to control Erk1/2 phosphorylation^24^ or the Cdc42 effector PAK7, associated with actin-modulated endothelial barrier integrity.^25^ However, the specific nature of the above phenomena remains to be elucidated.

We cannot discard that other molecular cues could be involved in the observed effects following Erk1/2 inhibition and/or cAMP elevation. For example, calcium (Ca^2+^) modulation may also be relevant because treatment with forskolin raises the concentration of intracellular Ca^2+.26^ In fact, cAMP, via either PKA or Epac1, has been proposed to modulate inositol triphosphate (IP_3_) production and IP_3_ receptors activation, thus promoting the release of Ca^2+^ from the endoplasmic reticulum.^27^ Although most studies have reported negative effects of Ca^2+^ on endothelial cell function (reviewed in^28^); few others have shown quite the opposite. For example, endothelial cell activation, as measured by ICAM-1 expression, provoked by different stimuli was prevented by pre-treatment with media containing high levels of Ca^2+^. In addition, Ca^2+^ supplementation also prevented endothelial activation mediated by trophoblastic debris obtained from preeclamptic placentae.^29,30^ Although, these studies did not explore endothelial barrier function, it is accepted that endothelial activation also involves local permeability and endothelial cell contraction. Thus, Ca^2+^ could be an attractive candidate to explain the Rac1-independent increase of barrier function reported in this study.

Lastly, we explored the effects of F/R and Erk1/2 inhibition in perivascular permeability *in vivo* using the well-established Miles assay. Intradermal injection of U0126 significantly induced permeability compared to DMSO- or F/R-application. Injection of F/R and U0126 combination, on the other hand, prevented the increased perivascular permeability caused by Erk1/2 inhibition. This finding is consistent with our *in vitro* data, where lower TEER resistance was observed as a result of U0126-mediated Erk1/2 inhibition but application of either F/R or F/R+U0126 led to resistance enhancement. Our findings are also in line with other studies showing that Erk1/2 plays a critical role in maintaining the integrity of the endothelium *in vivo*^3^; and that Erk1/2 activation was beneficial for endothelial barrier function and homeostasis in a MEK/Erk-KCa3.1-dependent fashion.^31^ Clearly, Erk1/2 is an attractive target for endothelial barrier modulation. On the other side, our data are in contrast to previous studies showing that activation of Erk1/2 pathway occurs after oxygen–glucose deprivation and is detrimental for cerebral endothelial cells viability^32^ or promotes diffuse alveolar hemorrhage and lung injury in B6 mice subjected to pristane treatment, a natural saturated terpenoid alkane used to induce autoimmunity in rodents.^33^ Particularly, injury in cerebral endothelial cells was enhanced by treatment with 10 µM and not with 1 µM U0126, indicating that inhibitor dosage is relevant and must be considered when comparing outcomes from different models. Furthermore, the complete deletion of Erk1 or Erk2 in mice as seen in,^3^ could also have an off-target effect in molecules not investigated that contribute to the phenotypes observed. Besides, living animals are a more complex system where several cell types coexist and can trigger effects between each other. Nevertheless, it is clear that Erk1/2 exerts diverse functions depending on the cell type or animal model used. This is not surprising, since different cell types and even endothelial cells from distinct vascular beds have heterogeneous protein expression. In this regard, a recent study compared the RNAseq expression profile of endothelial cells from brain, lung and heart at baseline and upon inflammation and demonstrated the existence of a clear genetic footprint specific for each endothelial cell type.^34^ Moreover, the gut vascular barrier shows differential claudin-7, −12, −15 and −34c1 expression when compared to brain endothelium (reviewed in detail^35^). The findings in the current study are limited to heart endothelium and skin vasculature. The contribution of other factors, such as cell immortalization, blood flow, extracellular matrix components and other cell types, remains to be explored in the context of Erk1/2 inhibition. Therefore, extrapolating the effects caused by Erk1/2 inhibition described here must be done considering that they might be specific to the cardiac endothelium. Thus, future efforts should focus on investigating the tissue-specific mechanisms exploited by Erk1/2 that are relevant for endothelial barrier function.

In summary, our data show that Erk1/2 is involved in the regulation of endothelial barrier function and intervenes with cAMP-dependent Rac1 activation without affecting the barrier stabilizing effects induced by cAMP.

## Data Availability

The data that support the findings of this study are available from the corresponding author, AGP, upon reasonable request.
